# Impact of body characteristics on ultrasound-measured inferior vena cava parameters in Chinese children

**DOI:** 10.1590/1414-431X20198122

**Published:** 2019-09-16

**Authors:** Jianjun Gui, Boyang Zhou, Juanhua Liu, Bing Ou, Yue Wang, Longyuan Jiang, Wanchun Tang, Baoming Luo, Zhengfei Yang

**Affiliations:** 1 Department of Emergency Medicine, Shiyan People's Hospital of Bao'an District, Shenzhen, Guangdong, China; 2 Emergency Department of TungWah Affiliated Hospital, Sun-Yat Sen University, Dongguan, China; 3 Sun-Yat Sen Memorial Hospital, Sun Yat-sen University, Guangzhou, China; 4 The Eastern Hospital of the First Affiliated Hospital, Sun-Yat Sen University, Guangzhou, China; 5 Weil Institute of Emergency and Critical Care Medicine, School of Medicine, Virginia Commonwealth University, Richmond, VA, USA; 6 Zeng Cheng District People's Hospital of Guangzhou, Guangzhou, China

**Keywords:** Inferior vena cava, Abdominal aorta, Body characteristics, Volume status

## Abstract

Ultrasound-measured inferior vena cava (IVC) and abdominal aorta (Ao)-associated parameters have been used to predict volume status for decades, yet research focusing on the impact of individual physical characteristics, including gender, height/weight, body surface area (BSA), and age, assessed simultaneously on those parameters in Chinese children is lacking. The aim of the present study was to explore the impact of individual characteristics on maximum IVC diameter (IVCmax), Ao, and IVCmax/Ao in healthy Chinese children. From September to December 2015, 200 healthy children from 1 to 13 years of age were enrolled. IVCmax and Ao diameters were measured by 2D ultrasound. We found that age (years), height (cm), weight (kg), waist circumference (cm), and BSA (m^2^) were positively correlated with IVCmax and Ao. Multivariate linear regression showed that age was the only independent variable for IVCmax (mm) in female children, height was the only independent variable for IVCmax in male children, and age was the only independent variable for Ao in both females and males. IVCmax/Ao was not significantly influenced by the subjects’ characteristics. In conclusion, IVCmax and Ao were more susceptible to subjects’ characteristics than IVCmax/Ao. IVCmax/Ao could be a reliable and practical parameter in Chinese children as it was independent of age, height, and weight.

## Introduction

Diarrheal diseases and associated complications are major worldwide health threats to children, especially children under the age of 5 (1). Dehydration is one of the most common complications of diarrheal diseases. The American College of Critical Care Medicine emphasized the importance of early and aggressive fluid resuscitation and early evaluation of volume status in the setting of septic shock (2–5). However, there is no reliable method to objectively measure the degree of dehydration and to guide fluid resuscitation. Sensitivity and specificity are limited in both signs- and symptoms-derived clinical scales, such as the World Health Organization (WHO) scale, the Gorelick scale, the clinical dehydration scale (CDS), and lab values (6–9).

Sonographic inferior vena cava (IVC) parameters have been used to guide fluid management in adults for decades because of their unique features, including safety, rapidity, non-invasiveness, and portability (10–13). In recent years, ultrasound-measured IVC parameters have been gradually and widely used in children. The inferior vena cava/abdominal aorta ratio (IVCmax/Ao) was first introduced to identify severe dehydration in 2007 by Chen (14), and it was later validated by other two studies in 2010 (10,15). These two studies pointed out that an IVCmax/Ao of less than about 1.2 does not indicate severe dehydration in children with symptoms of gastroenteritis. However, Levine et al. (15) found that although IVCmax/Ao was associated with volume status, it was not accurate enough to predict dehydration in children under 5 years of age. Different results might be related to different age groups, and studies focusing on the impact of age on IVCmax/Ao are rare, especially in Chinese children.

The aim of the present study was to explore the impact of individual characteristics on IVCmax/Ao, and IVC and Ao diameter in healthy Chinese children.

## Material and Methods

### Study design

This cross-sectional descriptive study was designed based on the Declaration of Helsinki II and was approved by the Medical Ethics Committee of TungWah Affiliated Hospital of Sun-Yat Sen University. A statement of informed consent was obtained. Participants' guardians were notified of the aim and procedures of this study by reading the consent form before signing the informed consent. The measurement was performed after the assent by the children.

### Study setting and population

From September to December 2015, we recruited 200 healthy children aged from 1 to 13 years. Participants lived in the local community and were visiting TungWah Affiliated Hospital for routine comprehensive health check-ups during the study enrollment period. Exclusion criteria included: age over 13 years, dyspnea, pyrexia, dehydration, and other obvious signs of unhealthiness, fasting period of less than 4 h, poor quality imaging due to patient's limited acoustic window, and lack of parental approval. Neither the children nor their guardians received remuneration for their participation in the present study.

### Study protocol

The demographic information of participants was obtained, including age, gender, and history of congenital disease. The anthropometric measurements were performed in each participant with light clothes and no shoes. Waist circumference was measured midway between the lower rib and the iliac crest, while the participant was standing erect with relaxed abdominal muscles. The tape was non-extensible and positioned horizontally and paralleled to the floor. Then, weight was measured on a scale. Body surface area (BSA) was calculated using the DuBois formula: BSA = (W^0.425^ × H^0.725^) × 0.007184.

In order to achieve consistent fluid status, participants were asked to fast for at least 4 hours before undergoing IVC and Ao ultrasound (US). US was performed using a Toshiba Aplio 500 Ultrasound System (Toshiba Medical Systems Corporation, Japan), equipped with 3.5 MHz convex probe. The IVC diameter (IVCmax) was measured with the transducer placed inferior to the xiphoid, and in order to obtain the long axis image of the IVC, the transducer was processed along the midline. The measurement procedure was recorded in 15 s B-mode (grayscale) video clips to ensure that respiratory variations were accounted for and the points of IVCmax were captured. The measurement of IVCmax was performed just proximal to the hepatic veins, where the anterior and posterior walls of the IVC are easily seen and parallel to each other during the end exhalation period. The Ao diameter was taken in a similar manner during systole, 3 to 5 mm above the celiac trunk, from one interior wall to the opposite interior wall. All the US measurements were performed by an ultrasound specialized physician with more than 10 years of experience in cardiovascular US.

### Data analysis

Data are reported as means±SD and 2 SD range or proportions, whichever was appropriate. Normality of continuous variables was assessed by histogram plots and the one-sample Kolmogorov-Smirnov test with P>0.05 indicating an adequate approximation of normal distribution. Pearson coefficients were obtained from correlation tests between continuous variables, with transformations used before analysis, as necessary. Multivariate linear regression (forward selection with likelihood ratio criterion for selection variables: P<0.05 to enter, ≥0.05 to remove) was used to test the independent association of previously correlated variables with the IVCmax and Ao. All statistical analyses were performed using Statistical Package for Social Sciences version 19.0 (SPSS, IBM, USA). All P values were two-tailed, and those <0.05 were considered statistically significant.

## Results

### Demographic data

The relevant individual characteristics of the 200 children and IVCmax, Ao, and IVCmax/Ao are shown in Table 1.

**Table 1.** Comparison of age, body size parameters, IVCmax, and Ao between males and females.

	Total (n=200)	Gender		P for gender
		Female (n=92)	Male (n=108)	
Age (years)	6.87±3.41	6.34±3.35	7.32±3.42	0.041
Height (cm)	117.00±20.25	113.40±20.18	120.10±19.88	0.020
Waist (cm)	55.16±7.83	53.55±7.39	56.53±7.96	0.007
Weight (kg)	22.69±8.10	21.21±7.64	23.94±8.31	0.018
BSA (m^2^)	0.85±0.23	0.81±0.23	0.89±0.24	0.020
IVCmax	10.06±2.42	9.75±2.42	10.33±2.40	0.090
Ao	9.84±2.26	9.50±2.26	10.12±2.22	0.052
IVCmax/Ao	1.04±0.18	1.04±0.19	1.04±018	0.769

BSA: body surface area; IVCmax: maximum inferior vena cava diameter; Ao: abdominal aorta diameter.

### IVCmax diameter, Ao diameter, and the IVCmax/Ao index

**Figure 1.**
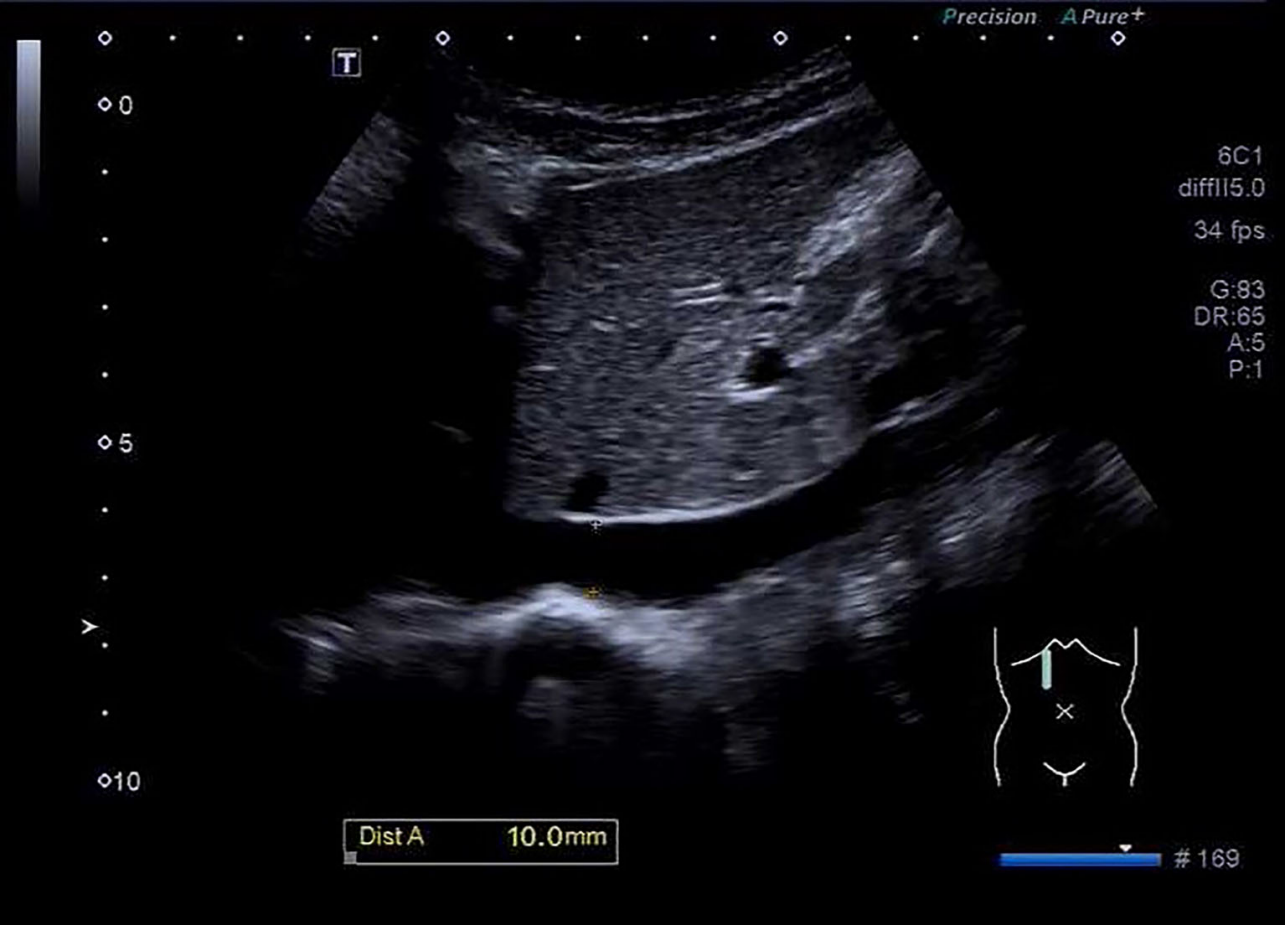
Longitudinal view of the maximal inferior vena cava diameter (10 mm) during expiration through the liver in a 9-year-old boy.

**Figure 2.**
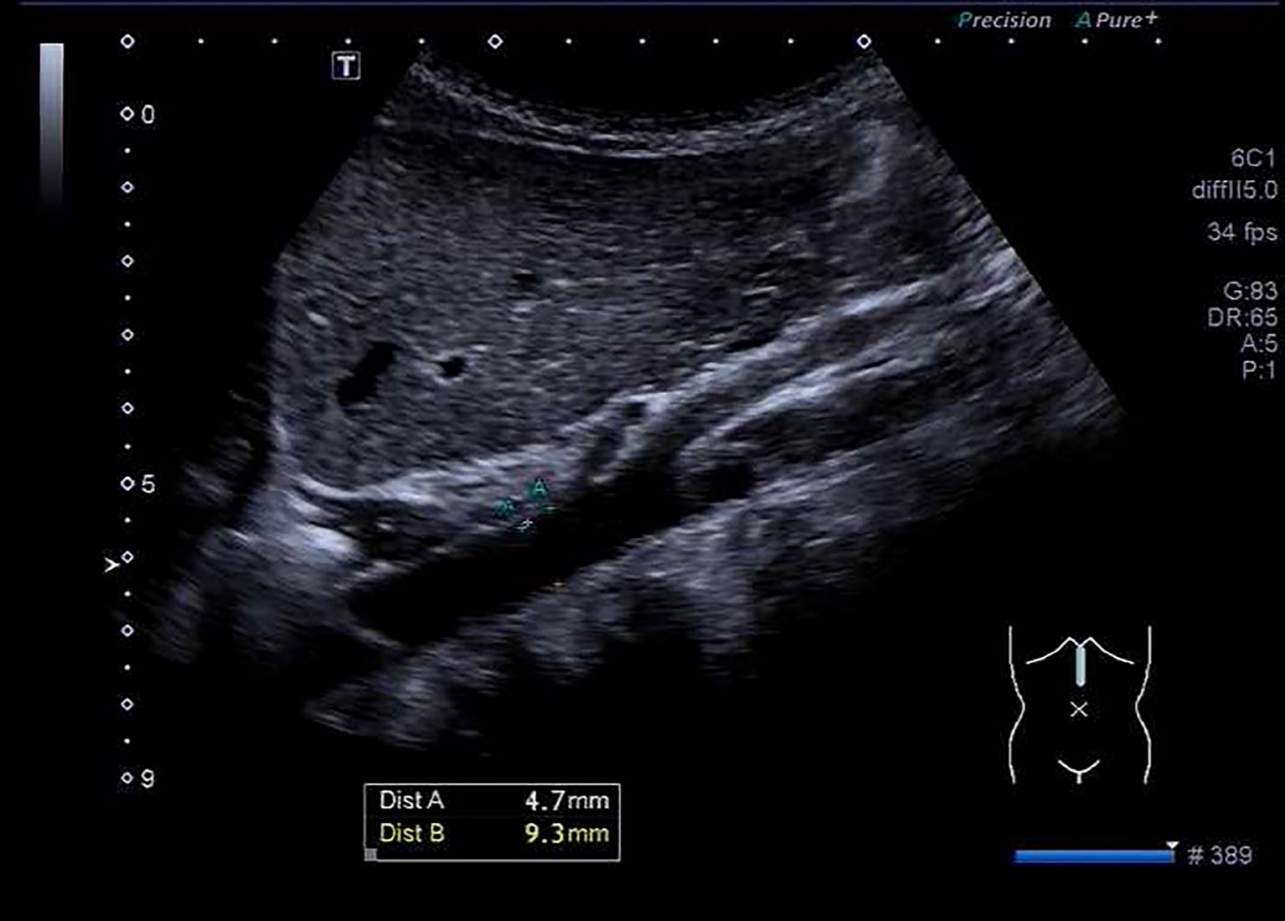
Longitudinal view of the aorta (Ao) during systole in the same boy of Figure 1. The Ao diameter of 9.3 mm (Dist B) was measured during systole and 4.7 mm (Dist A) above the celiac trunk from one interior wall to the opposite interior wall.

The measurements of IVC and Ao are presented in Figures 1 and 2. Table 2 shows the correlations between individual characteristics and IVCmax, Ao, and the IVCmax/Ao index. Briefly, age, height, weight, waist, and BSA were positively correlated with IVCmax and Ao. Age, height, weight, waist, and BSA were negatively correlated with IVCmax/Ao among all participants and females, while age, height, weight, and BSA were negatively correlated with IVCmax/Ao only among males.

**Table 2.** Correlation coefficients between body characteristics and maximum inferior vena cava diameter (IVCmax), abdominal aorta diameter (Ao), and IVCmax/Ao.

	Total (n=200)	Male (n=108)	Female (n=92)
IVCmax			
Age (years)	0.71***	0.73***	0.67***
Height (cm)	0.70***	0.76***	0.61***
Waist (cm)	0.50***	0.49***	0.48***
Weight (kg)	0.67***	0.69***	0.64***
BSA (m^2^)	0.69***	0.74***	0.63***
Ao			
Age (years)	0.87***	0.85***	0.89***
Height (cm)	0.82***	0.80***	0.83***
Waist (cm)	0.51***	0.49***	0.52***
Weight (kg)	0.80***	0.76***	0.84***
BSA (m^2^)	0.82***	0.79***	0.84***
IVCmax/Ao			
Age (years)	-0.17*	-0.10	-0.24*
Height (cm)	-0.12	-0.002	-0.25*
Waist (cm)	-0.002	0.04	-0.05
Weight (kg)	-0.13	-0.04	-0.22*
BSA (m^2^)	-0.12	-0.02	-0.24*

BSA: body surface area. *P<0.05, ***P<0.001.

### Regression models

To determine the independent influence of individual characteristics on IVCmax in females I (Supplementary Table S1), three regression models were used. Multiple regression model 1 had IVCmax as a dependent variable, and age as a sole independent variable. Model 2 was similar but height and weight were added as independent variables. Lastly, model 3 used BSA as an independent variable instead of height and weight to test whether BSA improved the statistical stability and goodness of fit of the model. Model 1, model 2, and model 3 had similar multiple R values (0.45, 0.44, and 0.44, respectively). After adjusting for confounders, age (years) was the only independent variable for IVCmax (mm) in females (β=0.49, P<0.001); neither BSA nor "height and weight" independently influenced IVCmax in females.

However, in the three similar multiple regression models used to test independent variables for IVCmax in males (Supplementary Table S1), height (mm) was the only significant independent variable for IVCmax (β=0.08, P=0.001). The entire model accounted for 58% of the variability in the IVCmax (adjusted R^2^=0.58).

The independent variables for Ao were also tested with the aforementioned three multiple regression models (Supplementary Table S2), and age (years) was the only independent variable for Ao in both females and males (β=0.6, P<0.001 and β=0.55, P<0.001, respectively). The model accounted for 80% of the variability in the Ao in females (adjusted R^2^=0.8) and 72% in males (adjusted R^2^=0.72).

The regression model of IVCmax/Ao depicted that IVCmax/Ao was not substantially influenced by the subjects’ characteristics (Supplementary Table S3).

## Discussion

We investigated the impact of individual characteristics on IVCmax/Ao, IVCmax, and Ao diameter in 200 healthy Chinese children. In our previous study, we investigated the impact of individual characteristics on collapsibility index (CI) in adults and found that its ability to detect intravascular volume was influenced by age, BMI, and baseline CI (16). However, the evidence concerning the impact of individual characteristics on IVCmax, Ao, and IVCmax/Ao in children was limited.

Taneja et al. (17) correlated the US measurement of IVC solely with the subjects' height, weight, and BSA in Indian children. In that study, the US measurement was performed by different investigators following the same methodology with M-mode imaging. Nevertheless, different investigators could reduce consistency, and the acquisition of reproducible M-mode measurements could be easily affected by natural movement of the diaphragm. In another study, Hegde et al. (18) investigated aorta diameter standards at multiple levels using computed tomographic data in healthy children. In their linear regression analysis, BSA was evaluated instead of weight and height. However, using BSA instead of height and weight might decrease the statistical stability and accuracy of the model (19). To the best of our knowledge, there is no published study focusing on the impact of individual characteristics on IVCmax/Ao in children, especially in Chinese children.

The impact on IVCmax and IVCmax/Ao in adults has been previously studied by Gui et al. (20). Comparatively, the impact of individual characteristics on IVCmax and Ao were significantly stronger in children than in adults (R^2^=0.517, R^2^=0.079 for IVCmax and R^2^=0.759, R^2^=0.256 for Ao in children and adults, respectively). In contrast, the impact on IVCmax/Ao was weak in both children and adults (R^2^=0.039, R^2^=0.005 for female and male children, R^2^=0.123 for Ao in adults). Similarly to our study, Stenson et al. (21) investigated the normative of IVC and Ao measurements in children younger than 6 years and found that both IVC and aortic dimensions increased in a linear fashion and had excellent correlations with the body surface area, body mass, and height. Our study first found that IVCmax/Ao is not susceptible to individual patient characteristics in children aged 1 to 13, though IVCmax and Ao are susceptible to individual patient characteristics. Additionally, in dehydrated children, IVCmax decreased with decreasing blood volume, while Ao diameter remained mostly constant, despite intravascular volume depletion (22). Comparing to IVCmax, IVCmax/Ao might be more reliable to evaluate the dehydration level as it is not susceptible to individual patient characteristics in children aged 1 to 13. In our study, multivariate linear regression model showed that age was the only independent variable for IVCmax in female children, height was the only independent variable for IVCmax in male children, and IVCmax/Ao was not substantially influenced by the subjects’ characteristics. Adults and children are of different physical statures, which indicates that as age and height increase over time, the diameter of IVC and Ao change as well (Supplementary Tables S1 and S2). This physical difference between children and adults might be the reason why IVCmax/Ao is susceptible to adult patients’ characteristics but not to children's. In view of the fact that IVCmax/Ao is less affected by individual characteristics, it could be a reliable and practical metric without considering the impact of individual age, height, and weight on IVCmax. Our study also established the reference ranges for IVCmax (5.22–14.9 mm), abdominal Ao (5.32–14.36mm), and IVCmax/Ao (0.68–1.4) in healthy Chinese children.

There are some limitations in this study. Firstly, our data were collected in a single center in China. Thus, the sample size of our study was limited and may not apply to all Chinese children. Secondly, the volunteers enrolled in our study were physically active, healthy, and were visiting for routine comprehensive health check-ups, which might have led to some selection bias. Therefore, we have no long-term organ function or outcome data and the IVCmax/Ao relationship in children with dehydration or fluid resuscitation. However, the data in our study could serve as normal control values to guide clinical practice in Chinese children with diarrhea, dehydration, or other diseases.

## Supplementary Material

Click here to view [pdf].
